# Direct observation of potential phase at joining interface between *p*-MgO and *n*-MgFe_2_O_4_

**DOI:** 10.1038/s41598-020-73849-9

**Published:** 2020-10-13

**Authors:** Chisato Sakaguchi, Yasumasa Nara, Takeshi Hashishin, Hiroya Abe, Motohide Matsuda, Sadahiro Tsurekawa, Hiroshi Kubota

**Affiliations:** 1 Graduate School of Science and Technology, Kumamoto University, 2-39-1 Kurokami, Chuo-ku, Kumamoto, 860-8555 Japan; 2 Division of Materials Science and Chemistry, Faculty of Advanced Science and Technology, Kumamoto University, Kurokami, Chuo-ku, Kumamoto, 860-8555 Japan; 3 Joining and Welding Research Institute, Osaka University, 11-1 Mihogaoka, Ibaraki, Osaka 567-0047 Japan

**Keywords:** Materials science, Nanoscale materials, Electronic properties and materials

## Abstract

Visualization of the depletion layer is a significant a guideline for the material design of gas sensors. We attempted to measure the potential barrier at the interface of core–shell microspheres composed of *p*-MgO/*n*-MgFe_2_O_4_/Fe_2_O_3_ from the inside out by means of Kelvin probe force microscopy (KPFM) as a first step to visualizing enlargement of the depletion layer. As determined by high-angle annular dark-field scanning transmission electron microscopy, ca. 70% of the microspheres were hollow with a wall thickness of ca. 200 nm. Elemental mapping revealed that the hollow particles were composed of ca. 20 nm of MgO, ca. 80 nm of MgFe_2_O_4_, and ca. 100 nm of Fe_2_O_3_. A difference of 0.2 V at the *p*-MgO/*n*-MgFe_2_O_4_ interface was clarified by KPFM measurements of the hollow particles, suggesting that this difference depends on the formation of a *p*–*n* junction. The potential barrier enlarged by the formation of a *p*–*n* junction was considered to increase the resistance in air (*R*_a_), since the *R*_a_ of the core–shell hollow microspheres was higher than that of MgO, Fe_2_O_3_, MgO–Fe_2_O_3_, and MgO/MgFe_2_O_4_/Fe_2_O_3_ particles with irregular shapes. Measurement of the potential barrier height by KPFM is a promising potential approach to tuning the gas sensitivity of oxide semiconductors.

## Introduction

The detection of various gases is important for constructing safety nets in today’s society. For example, one type of well-known detection technology is monitoring systems for gas leaks and flammable gases such as methane that originates from coal oil complexes^1^. Oxide semiconductors are used for these gas detection method^2,3^, wherein the presence of a gas is detected based on a change in resistance caused by the electrical interaction between gas molecules and the oxide semiconductor^4,5^. For gas detection with oxide semiconductors, a linear relationship between the change in electrical resistance and gas concentration is assumed as a rule of thumb. According to assumption, increasing the amount of adsorbed oxygen is key to enhancing the sensor response to gas, or gas sensitivity. Adsorbed oxygen is generated when oxygen in the air takes electrons from the surface of the oxide semiconductor and adsorbs to the surface as negatively charged species (adsorbed oxygen: O^−^, O^2−^). At the same time, a space charge layer (depletion layer) with a low carrier concentration forms from the surface of the oxide inward^6^. Controlling this depletion layer could allow for tuning of the gas sensitivity.

Oxide semiconductor sensors using a change in a depletion layer enlarged by formation of a *p*–*n* junction can be categorized following: the enlargement of depletion layer originates only from *p*-*n* junction^7–15^; that from sulfurization on the surface of *p*-type oxide. Especially, latter contains electronic sensitizing by sulfurization^16–20^, as shown in Table 1. Among the former, the morphology of *n*-TiO_2_/*p*-CuO nanowires was effective to enhance sensor response to 1 ppm CO as one of reductant gases when the wall thickness of TiO_2_ shell was 60 nm. This result was supported by the experimental fact that its base resistance in air was highest in the wall thickness from 0 to 100 nm. If there is correlation data between the combination of *p*–*n* junction and the enlargement of depletion layer, the sensor with high sensing performance could be designed by selecting the optimal combination of *p*–*n* junctions without try and errors of experiments.

**Table 1:** Oxide semiconductor sensors using a change in a depletion layer enlarged by formation of a *p*–*n* junction.

Materials	Morphology	Target gases	Operating temperature (°C)	Comparison of sensor response		p–n junction effect (Ratio of composite to pure in sensor response)	References
				Pure	Composite		
NiO/ZnO	Nanosheet	100 ppm TEA	320	78.4(ZnO)	185.1	2.36	^7^
NiO/ZnO	Hierarchical flower-like	100 ppm acetone	330	< 7(ZnO), < 3(NiO)	< 12	< 1.7(ZnO), < 4(NiO)	^8^
NiO/ZnO	Nanofiber	10 ppm H2	200	n/a	60%(S = ΔR/Ro × 100)	n/a	^9^
NiO/SnO2	Hollow sphere	10 ppm TEA	220	14.5(SnO2)	48.6	3.35	^10^
CuO/ZnO	Microcube	50 ppm ethanol	240	≒ 2.5(CuO)	4.5	1.8	^11^
CuO/TiO2	Thin film/nanotube	1000 ppm H_2_	200	0.26(CuO), 0.88(TiO2)	2(S = ⊿I/Io)	8(CuO), 2(TiO2)	^12^
107950039243000TiO2/CuO	Nanowire	1 ppm CO	300	1.1(CuO)	7.14	6.5	^13^
SnO2-NiO	Powder paste	1000 ppm ethanol	150	n/a	84.7	n/a	^14^
SnO2/CuO	Nanowire	50 ppm HCHO	250	≒1.4(CuO)	≒ 2.4	1.7	^15^
CuO-SnO2	Powder paste	50 ppm H_2_S	200	7.8(SnO2)	35,000	4487	^16^
CuO-SnO2	Powder paste	200 ppm H_2_S	≒77	n/a	< 800	n/a	^17^
CuO/In2O3	Octahedral-like	10 ppm H_2_S	200	≒60(CuO)	≒ 120	2.0	^18^
107632526162000CuO-ZnO	Hollow tubule	50 ppm H_2_S	170	n/a	< 65	n/a	^19^
CuO/ZnO	Hollow nanofiber	100 ppm H_2_S	250	10(ZnO), 1.3(CuO)	< 60	6(ZnO), 46(CuO)	^20^

"/" denotes outside oxide/inside oxides, and "–" mixtures.

The enlargement of depletion layer originates only from *p*–*n* junction is show in Refs.^7–15^, and that from sulfurization on the surface of *p*-type oxide in Refs.^16–20^.

As a first step toward this goal, this study visualized the increase in the depletion layer due to *p*–*n* junction formation by measuring the barrier height at the grain boundary from the local surface potential near the grain boundary using a Kelvin probe force microscope (KPFM). The first visualization of the depletion layer is the measurement of the surface potential of the silicon *p–n* junction by KPFM^21^. After that, it was reported that the barrier height at the grain boundary of polycrystalline silicon was measured by using KPFM, and the potential barrier height changed depending on the grain boundary character^22^. As a recent visualization technique of the depletion layer, when the forward bias and the reverse bias were applied to the operating semiconductor material (GaAs), the change of the depletion layer width of only 1 nm was measured^23^. Besides, the electron beam induced current (EBIC) realized a visualization of the depletion layer by two-dimensional mapping^24^. However, advanced thinning technique is required to visualize the depletion layer by above techniques.

**Figure 1:**
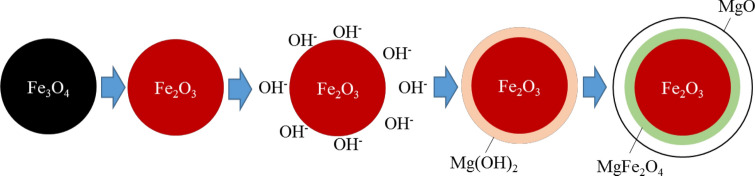
Preparation of core–shell microspheres. The Fe_2_O_3_ cores were completely covered with MgO shells, and the interfacial MgFe_2_O_4_ phase was formed by heating at 800 °C for 3 h.

In this study, it is possible to form heterogeneous junctions by our simple process (Fig. 1) for preparation of core–shell micro hollow particles. The KPFM measurement sample can also be prepared with a simple method (embedding the particles in a conductive resin and polishing them).

In our previous research, we revealed that composite oxides of MgO–Fe_2_O_3_ respond to 10 ppb hydrogen sulfide^25^. It was found that MgO acts as a *p*-type semiconductor and MgFe_2_O_4_ acts as an *n*-type semiconductor, thereby contributing to an increase in the depletion layer due to the *p*–*n* junction. In this study, KPFM measurements clarified the formation of *p*–*n* junctions in core–shell microspherical particles with Fe_2_O_3_ as the core and MgFe_2_O_4_ and MgO as the shells. In addition, microstructural observations indicated that the MgO shell behaves as a semiconductor.

The preparation of core–shell microspherical particles is depicted (Fig. 1). After chemically attaching OH groups to the surface of hematite (Fe_2_O_3_) particles obtained by thermal oxidation of magnetite (Fe_3_O_4_) at 800 °C for 3 h, the particles were immersed in a solution containing magnesium ions to precipitate Mg(OH)_2_. By heat-treating (800 °C, 3 h), Mg(OH)_2_ was dehydrated to MgO, and MgFe_2_O_4_ was formed at the interface between the Fe_2_O_3_ core and MgO shell.

## Results and discussion

The X-ray diffraction (XRD) pattern of the obtained core–shell microspherical particles is shown in Supplementary Fig. S1. All patterns were attributed to the Fe_2_O_3_ core and MgFe_2_O_4_ and MgO shells. Since the diffraction peaks of MgFe_2_O_4_ and MgO partially overlap, the abundance was estimated as follows from the relative peak intensity ratio in the ICDD database.

1 $$ {\text{Abundance ratio }} = \, \Sigma I\left( {{\text{MgFe}}_{{2}} {\text{O}}_{{4}} } \right) \, / \, \Sigma I\left( {{\text{MgO}}} \right) $$

According to Eq. (1), the abundance ratio of the sample after heat treatment (800 °C, 3 h) was approximately 1, indicating the presence of the same amounts of the MgFe_2_O_4_ and MgO shell constituents.

The secondary electron image of the core–shell microspherical particles and the MgKα, FeKα, and OKα mapping images are shown in Supplementary Fig. S2. The diameter of the core–shell microspherical particles was approximately 1 μm, and Mg and Fe existed in a spherical shape.

**Figure 2:**
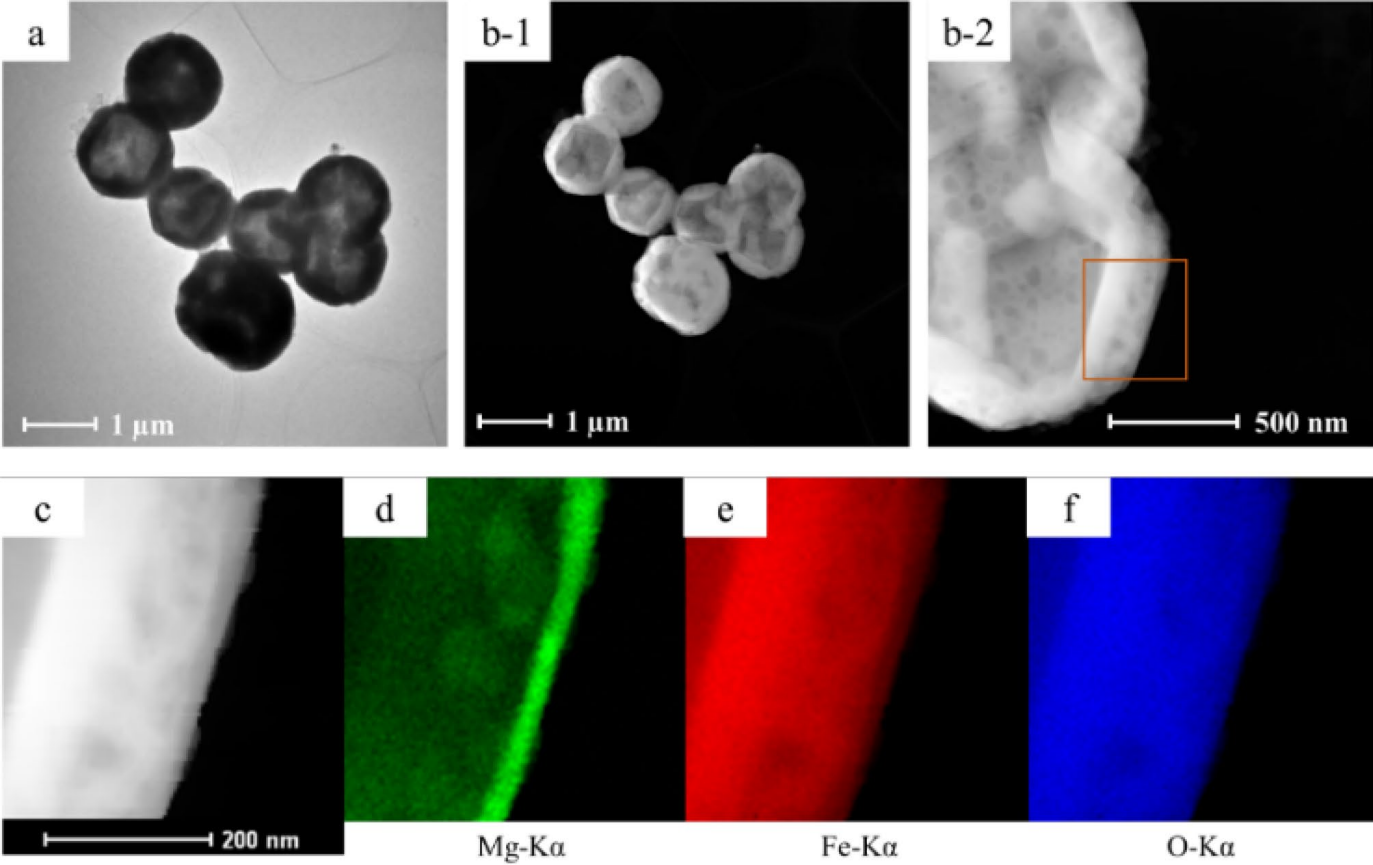
Microstructure of core–shell microspheres: (**a**) bright-field image obtained by inserting the object aperture into the center of diffraction spot, i.e., 000; (**b**) HAADF-STEM image; and elemental mapping images of (**c**) Mg-Kα, (**d**) Fe-Kα, and (**e**) O-Kα from the square area marked in (**b**).

The microstructural observations of the core–shell microspherical particles are shown in Fig. 2. The typical bright-field image shows the presence of hollow spherical particles (Fig. 2a). The abundance of hollow particles was approximately 70%, as indicated by the high-angle annular dark-field scanning transmission electron microscope (HAADF-STEM) images (Fig. 2b-1,2). Figure 2c is enlarged view of the square area in Fig. 2b-2. Based on the elemental mapping of the shell of the hollow particles (Fig. 2d–f), the shell thickness was approximately 200 nm. It is suggested that approximately 100 nm of the inner shell is Fe_2_O_3_, approximately 80 nm of the outer shell is MgFe_2_O_4_, and approximately 20 nm of the outermost surface is MgO. From these observations, it is assumed that the core Fe_2_O_3_ diffused into the shell MgO to form interfacial MgFe_2_O_4_, while Mg(OH)_2_ was dehydrated to MgO by the heat treatment (800 °C, 3 h).

**Figure 3:**
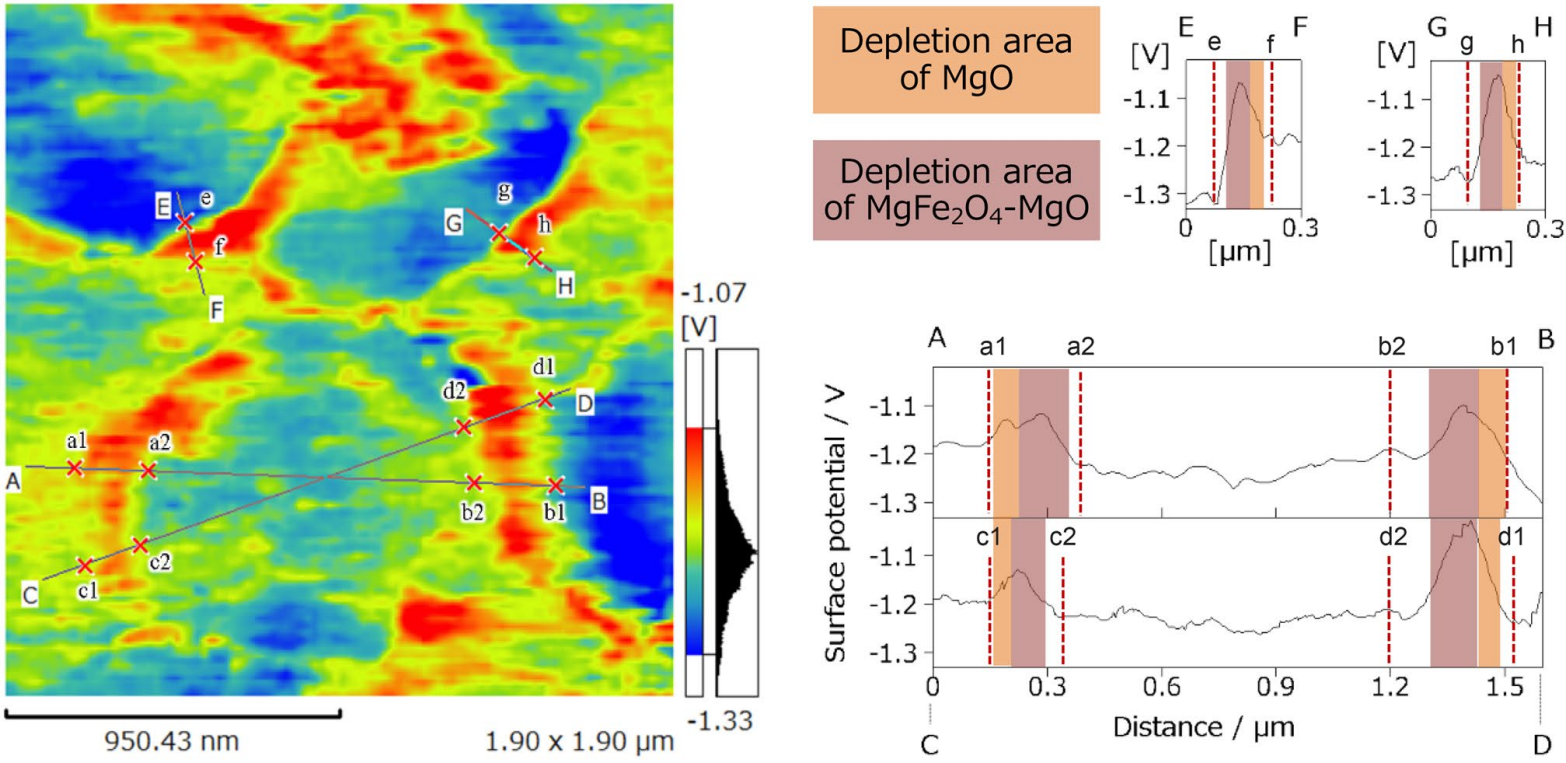
Potential barrier height of a core–shell microsphere cross-section measured by KPFM. The KPFM image was collected at a frequency of 0.1 Hz under an ambient atmosphere.

The KPFM results for the core–shell micro hollow particles are shown in Fig. 3. Since the core–shell particles were mirror-polished after being embedded in a conductive resin, the resin penetrated the hollow particles. The potential barrier of Cu within the conductive resin was adopted as the background potential for measuring the potential barrier height. In Fig. 3, high potential barriers are shown in red, and low are in blue. Based on the line analysis of four potential barrier heights (Fig. 3A–H), the average change in the potential barrier in the shell was approximately 0.2 V. In all four lines, the potential barrier in the inner shell tended to be higher than that in the outer shell. This may reflect the result of *p*–*n* junction formation between the *p*-type MgO and *n*-type MgFe_2_O_4_. Thus far, in the field of gas sensors, although it has been suggested that a *p*–*n* junction will cause the depletion layer to expand, this phenomenon has yet to be visualized.

**Figure 4:**
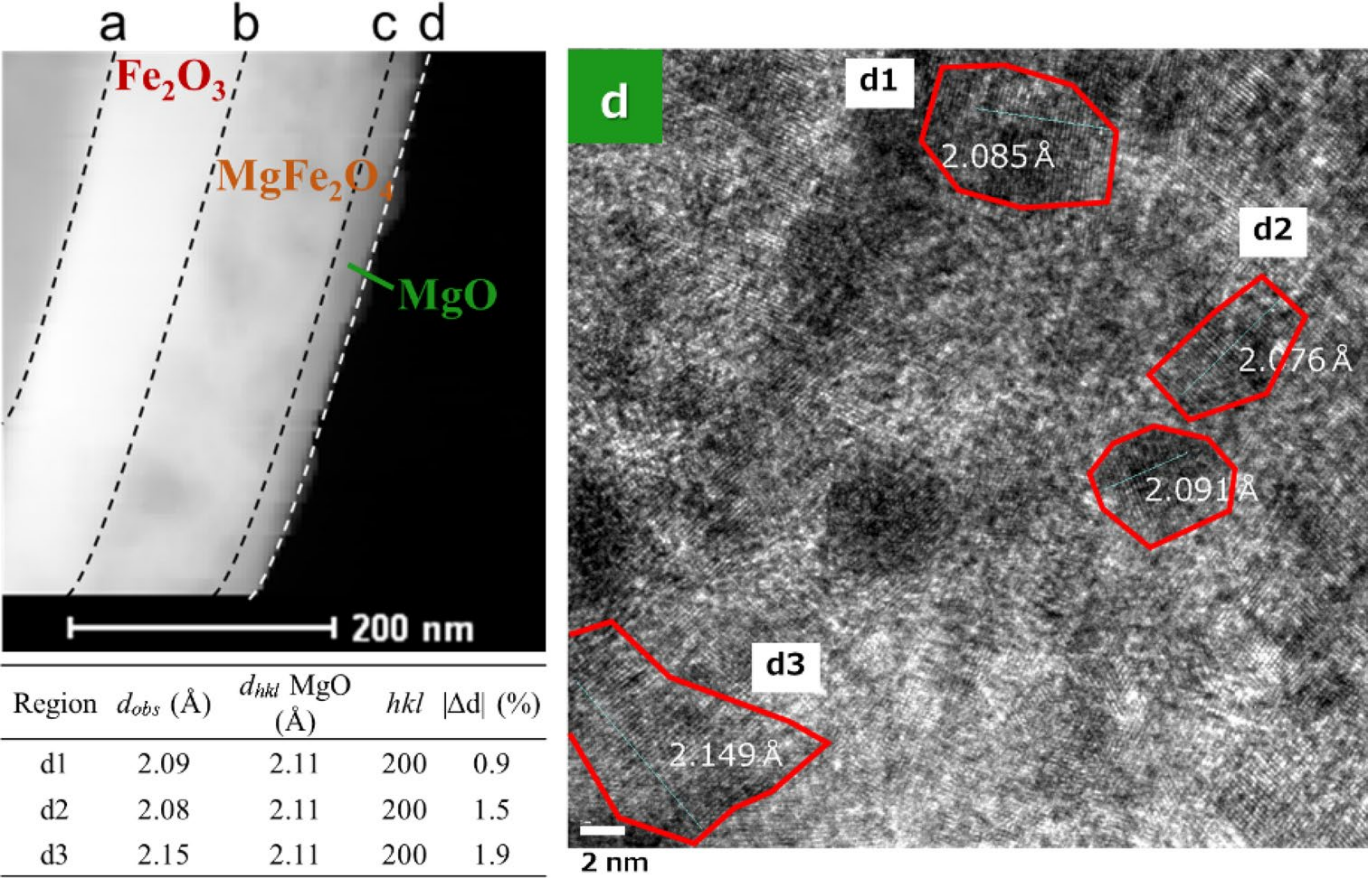
High-resolution TEM images of fine MgO crystals several nanometers in diameter that are mono-dispersed on the surface of the shell layer. Scale white bar is 2 nm.

In general, MgO is considered an insulator because of its high band gap (7.8 eV). However, recent studies have reported that one-dimensional MgO monolayers consist of an aggregate of several nanometers of MgO (MgO microcrystals) with a band gap of 3.2 eV^26^. Figure 4 shows a high-resolution TEM image of the MgO layer (approximately 20 nm) of the core–shell micro hollow particles in this study. The MgO layer consists of MgO microcrystals of several tens of nanometers, suggesting that the MgO layer in this study behaves as a semiconductor.

Supplementary Fig. S3 shows the temperature dependence of the electrical resistance in air (*R*_a_) of spherical Fe_2_O_3_ particles, amorphous MgO particles, a mixture of both particles, and the core–shell micro hollow particles. The core–shell micro hollow particles showed high *R*_a_ values at all temperatures due to the *p*–*n* junction effect.

The sensor response of MgO (Supplementary Fig. S3e) to 250 ppm at 3 ppm H_2_S was added as Fig. S4. The sensor response (S = Rg/Ra) increased upon exposure to H_2_S. Generally, in air, oxygen is adsorbed as a negative charge on the surface of an n-type oxide semiconductor (adsorbed oxygen: O^2−^), and a depletion layer is formed from the surface to the inside. When the atmosphere is switched from air to reducing gas (H_2_S), the adsorbed oxygen (O^2−^) reacts with H_2_S [H_2_S + 3 O^2−^ (ad.) ⇒ H_2_O + SO_2_ + 6e^−^], and the adsorbed oxygen electrons are depleted. By moving them to the layer, the depletion layer is reduced, leading to a reduction in electrical resistance. Since the behavior of the sensor response in Supplementary Fig. S4 showed an increase in resistance, the depletion layer is increasing. Therefore, it was considered that MgO behaved as p-type.

**Figure 5:**
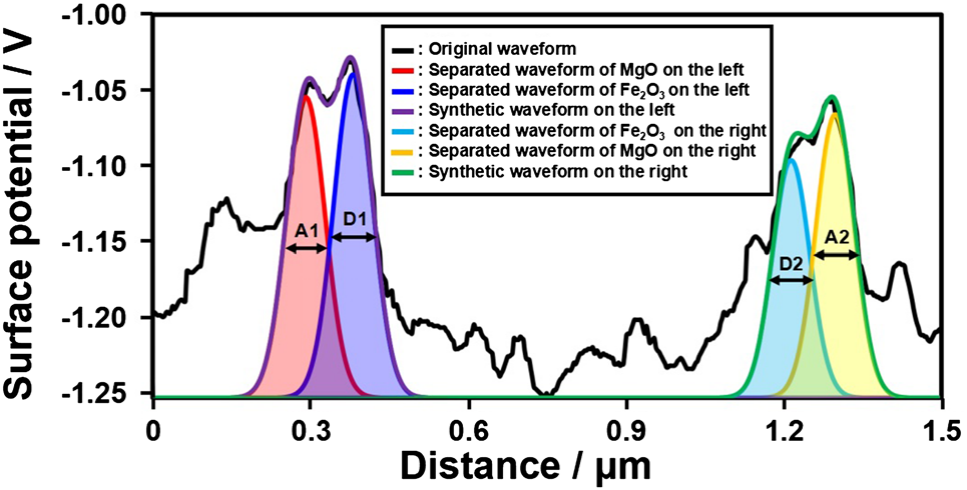
Waveform separation analysis of surface potential data at the *p*-MgO/*n*-MgFe_2_O_4_ interface. The symbols of A1, A2, D1, and D2 are respective full width half maximum (FWHM) of separated waveforms.

In order to investigate the cause of the difference in surface potential due to junction between oxides, the wave of surface potential in the same region as Fig. 3 was separated by Gaussian function. The width of depletion layer of each oxide was labelled as the full width half maximum (FWHM) in Fig. 5 in symbols of A1, A2, D1 and D2. Subsequently, the carrier concentration was calculated using the width. It was assumed that the dielectric constant of each oxide is proportional to the thickness of each oxide (MgO: 20 nm, MgFe_2_O_4_: 80 nm, Fe_2_O_3_: 100 nm) obtained from the HAADF-STEM image in Fig. 4. The dielectric constant of each region was calculated by distributing the dielectric constant of each oxide (MgO: 9.90^27^, MgFe_2_O_4_: 1541^28^, Fe_2_O_3_: 65.9^29^) based on the thickness of each oxide. The carrier concentration calculated using the dielectric constant of each region changed by the width of depletion layer at each region (Supplementary Table S1). Generally, the carrier concentration decreases as an enlargement of depletion layer width. The carrier concentration of MgO–MgFe_2_O_4_ (A1 and A2 of Supplementary Table S1) was higher than that of single crystal of MgO^30^ (3.6 × 10^17^ cm^–3^) and much lower than that of polycrystal of MgFe_2_O_4_^31^ (2.5 × 10^22^ cm^–3^). This is evidence that depletion layer was enlarged by *p*–*n* junction formation between the *p*-type MgO and *n*-type MgFe_2_O_4_. In the carrier concentration of MgFe_2_O_4_–Fe_2_O_3_ (D1 and D2 of Supplementary Table S1), same tendency was confirmed even considering the carrier concentration of Fe_2_O_3_^32^ (1.6 × 10^19^ cm^–3^). This suggests that the enlargement of depletion layer by formation of p–n junction could affect the carrier concentration of *n*–*n* junction (MgFe_2_O_4_–Fe_2_O_3_). From above discussion, it is considered that the KPFM measurement results of this study were effective to characterize electrically the core–shell microsphere particles.

## Conclusion

The MgO/MgFe_2_O_4_/Fe_2_O_3_ core–shell microsphere particles were prepared by heat treating core–shell microsphere particles with Fe_2_O_3_ as the core and MgO as the shell. Approximately 70% of the obtained spherical particles were hollow in structure and comprised, from the outside in, approximately 20 nm of MgO, 80 nm of MgFe_2_O_4_ and 100 nm of Fe_2_O_3_ from the outer shell. As determined by KPFM measurements of the hollow particles, the difference in potential barrier height at the interface between MgO and MgFe_2_O_4_ was approximately 0.2 V. This difference was reflected in the measured *R*_a_ values, suggesting that it was due to the formation of a *p*–*n* junction between *p*-type MgO and *n*-type MgFe_2_O_4_. In the development of gas sensors, measuring the potential barrier height with KPFM may lead to tunable gas sensitivity. As one of recent our results, the potential barrier height of joining interface between *p*-type CuO (Eg: 1.4 eV) and *n*-type SnO_2_ (Eg: 3.7 eV) was higher than that of CuO and SnO_2_. The sensitivity to detection gases could be tuned by selecting combination of *p*-type and *n*-type oxides among many oxide semiconductors with different bandgap, based on the magnitude in the resultant potential barrier height by the formation of *p*–*n* junction. The estimation of carrier concentration based on the surface potential of each junction of oxides measured by KPFM was clarified to be quite effective for depletion engineering of gas sensing materials.

## Methods

### Core–shell microspherical particle synthesis

Magnetite (Fe_3_O_4_) spherical particles with the diameter of ca. 1 µm were obtained by dissolving iron oxyhydroxide in a solvent (ethylene glycol and 9 wt% H_2_O) and hydrothermally treating it at 200 °C for 24 h in an autoclave, as described in our previously published work^33^. An Fe_2_O_3_ spherical powder with a particle size of 1 μm was then obtained by heat-treating (800 °C, 3 h). The Fe_2_O_3_ spherical particles were immersed in a 6 mol/L sodium hydroxide aqueous solution (alkalization). Subsequently, the alkalized Fe_2_O_3_ spherical particles were dissolved in an aqueous magnesium acetate (Mg(CH_3_COO)_2_) solution and stirred for 30 min. At this time, the molar ratio of Fe:Mg was 4:6. The obtained suspension was centrifuged (5000 rpm, 3 min) and heat-treated (800 °C, 3 h) to obtain core–shell microspherical particles.

### Sample for HAADF-STEM observation

The powder sample was dissolved in 10 mL of ethanol to 1 wt%, and a suspension was obtained through ultrasonication. The obtained suspension was dropped onto a Cu grid with a collodion film and dried to obtain the sample for TEM observation at 200 kV (TECNAI-F20, FEI, Japan). The core–shell microspherical particles were characterized by elemental mapping using an Si detector to detect characteristic X-rays (Mg-Kα: 1.253 keV; Fe-Kα: 6.398 keV; O-Kα: 0.525 keV).

### Sample for KPFM measurement

As a first step, a conductive resin (Technovit 5000, Kulzer, Germany) were prepared, which is composed of the mixture of powder (90 wt% Cu and 10 wt% benzoyl peroxide) and liquid (organic compounds: C_5_H_8_O_2_, C_12_H_18_O_4_, C_14_H_22_O_6_). The mixing weight ratio of powder to liquid is 2. The liquid was put in the half of the powder in a hard paper cup and mixed thoroughly. Continuously, the 2nd half of powder was poured in the cup after mixed for 40 s. After that, the mixture of the core–shell microspherical particles and the conductive resin were poured into a mold and cured to obtain the polishing sample. Parallel polishing (#1500, #2000, #2400, #4000) using water-resistant abrasive paper and buffing (diamond slurry: 3 μm, 0.25 μm) afforded the observation sample for KPFM with a mirror surface. The final shape of the sample was a disc of 10 mm diameter and 1 mm of thickness. Then, the sample was attached to the sample holder, which is stainless steel disk of 15 mm diameter and 1 mm thickness by carbon tape, and the lower surface of the holder was directly connected to the stage of KPFM (SPM-9700HT, Shimadzu, Japan).

### KPFM measurement conditions

The Kelvin Probe Force Microscopy (SPM-9700HT, Shimazu, Japan) was used for visualization of potential barrier height at the joining interface of MgO/MgFe_2_O_4_/Fe_2_O_3_ core–shell microsphere particles. The surface potential (in reality, the contact potential difference between the probe and sample) was measured by applying an alternating voltage between the probe and sample while vibrating the conductive probe at 0.1 Hz for square area of 2 µm × 2 µm and detecting the vibration of the cantilever at 74 kHz for KPFM mode due to electrostatic force. In this study, the sample was scanned with tapping mode by Pt–Ir coated Si cantilever with a curvature radius of tip, *R* ≤ 25 nm, resonance frequency, *f*_r_ = 75 kHz, force constant, *k*_f_ = 2.8 N/m (EFM-20, NanoWorld, Switzerland). The measurement was conducted with following conditions: scanning speed, 0.1 Hz; resolution, 256 × 256 pixels. The spatial resolution of this device is 0.2 nm. It is noted that the spatial resolution of this device is 0.2 nm.

### XRD measurement conditions

Powder XRD (Ultima IV, RIGAKU, Japan) was used to identify the crystalline phase of the core–shell microspherical particles. The tube voltage, tube current, and scanning speed were 45 kV, 40 mA, and 4°/min, respectively.

### Sample for SEM observation

Field emission scanning electron microscopy (JSM-7600F, JEOL, Japan) was used for morphological observations and elemental mapping of the core–shell microspherical particles. The accelerating voltage, working distance, and detection angle were 15 kV, 8 mm, and 15°, respectively.

### Sample for electrical resistance measurement

An Au wire was fixed with Au paste to an Au interdigitated electrode (teeth number: 50, gap size: 5 µm) via ordinary ultraviolet exposure, and the solvent was volatilized by heat treatment (800 °C, 3 h) to obtain an electrical resistance measurement substrate. After 2 μL of a 10 wt% aqueous suspension of core–shell microspherical particles was dropped onto the substrate and dried, the sample for electrical resistance measurement was obtained by heat treatment for ohmic contact (400 °C, 30 min).

## Supplementary information

Supplementary Information
