# Supplementary material for: Direct observation of potential phase at joining interface between p-MgO and n-MgFe2O4
Source: Sci Rep. 2020 Oct 13;10:17055. doi: 10.1038/s41598-020-73849-9 (PMC7555545; doi:10.1038/s41598-020-73849-9)
Supplement: Supplementary file 1 — Supplementary Information [file 41598_2020_73849_MOESM1_ESM.docx]

Direct Observation of Potential Phase

at Joining Interface between *p*-MgO and *n*-MgFe_2_O_4_

Chisato Sakaguchi^1^, Yasumasa Nara^1^, Takeshi Hashishin^2^, Hiroya Abe^3^, Motohide Matsuda^2^, Sadahiro Tsurekawa^2^, Hiroshi Kubota^1^

^1^Graduate School of Science and Technology, Kumamoto University, 2-39-1 Kurokami, Chuo-ku, Kumamoto 860-8555, Japan

^2^Division of Materials Science and Chemistry, Faculty of Advanced Science and Technology, Kumamoto University, Kurokami, Chuo-ku, Kumamoto 860-8555, Japan

^3^Joining and Welding Research Institute, Osaka University, 11-1 Mihogaoka, Ibaraki, Osaka 567-0047, Japan

Correspondence and requests for materials should be addressed to T. H. (e-mail: hashishin@msre.kumamoto-u.ac.jp)

Figure S1


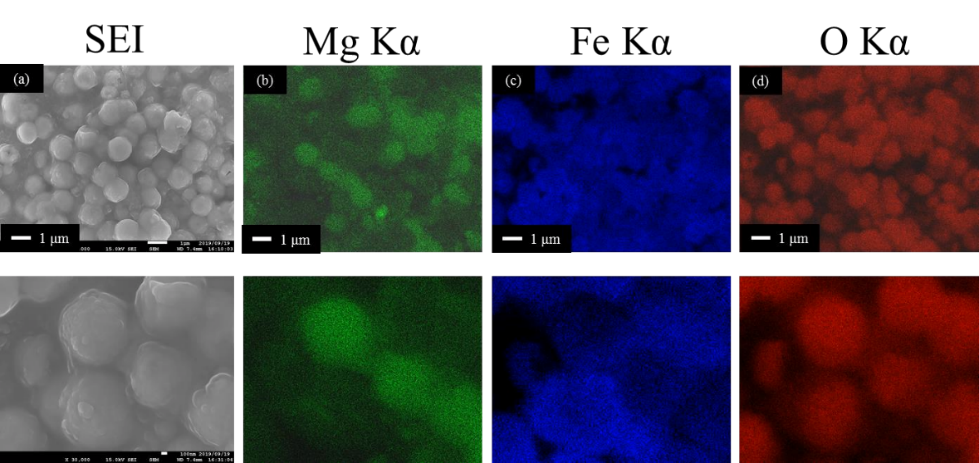


Figure S2

Figure S3

Air

Air

H_2_S

Figure S4

**Table S1**

**FIGURE CAPTIONS**

**Figure S1.** XRD pattern of core-shell microspheres. The bar graphs are based on the PDF database XRD patterns: MgFe_2_O_4_: 01-089-4924, MgO: 01-089-7746, and Fe_2_O_3_: 00-001-1053.

**Figure S2.** Microstructural features of core-shell microspheres: a) secondary electron image; elemental mapping images of b) Mg-Kα, c) Fe-Kα, and d) O-Kα.

**Figure S3.** Resistance in air (*R*_a_) of a) commercial MgO particles with irregular shape, b) core-shell microspheres composed of MgO, MgFe_2_O_4_ and Fe_2_O_3_, c) sintered particles composed of MgO, MgFe_2_O_4_ and Fe_2_O_3_ with irregular shape, d) sphere Fe_2_O_3_, and e) the MgO particles with irregular shape prepared from magnesium acetate. The *R*_a_ values were measured using Au interdigitated micro-electrodes.

**Figure S4.** Sensor response of MgO [Fig. S3 e)] to 3 ppm H_2_S at 250 ^o^C. The response value (S=*R*g/*R*a) was almost 1 after air flowed for 300 s, and increased after atmosphere changed from air to 3 ppm H_2_S. The S value almost closed to 1 after 300 s by changing from 3 ppm H_2_S to air.

**Table S1.** Depletion layer width of separated waveform and carrier concentration calculated using the dielectric constant of each depletion layer distributed by thickness of each oxide. Regions A1 and A2 correspond to MgO-MgFe_2_O_4_, and those D1 and D2 MgFe_2_O_4_-Fe_2_O_3_.
